# Development of a Novel Sandwich ELISA Test for the Detection of Antibodies Against Rickettsia

**DOI:** 10.3390/pathogens14121298

**Published:** 2025-12-18

**Authors:** Marco Quevedo-Diaz, Semen Kaliukanov, Frantisek Csicsay, Diana Hopkova, Ludovit Skultety

**Affiliations:** 1 Department of Rickettsiology, Institute of Virology, Biomedical Research Center, Slovak Academy of Sciences, Dúbravská Cesta 9, 84505 Bratislava, Slovakia; frantisek.csicsay@savba.sk (F.C.); diana.hopkova@savba.sk (D.H.); viruludo@savba.sk (L.S.); 2 Faculty of Natural Science, Department of Microbiology and Virology, Comenius University, Ilkovičova 3278/6, 84104 Bratislava, Slovakia; 3 National Reference Centre for Surveillance and Laboratory Diagnosis of Rickettsioses Banská Bystrica, Performance Workplace Institute of Virology, Dúbravská Cesta 9, 84505 Bratislava, Slovakia

**Keywords:** rickettsial infection, sandwich ELISA, *Rickettsia akari*, recombinant protein, GroEL, DnaK, A8GP63

## Abstract

Diagnosis of rickettsial infections is challenging due to nonspecific clinical symptoms and limitations of current diagnostic methods. Molecular assays allow early detection but are limited by cost and technical demands, whereas conventional serological tests often exhibit cross-reactivity and low sensitivity during the early stages of infection. This study aimed to develop and evaluate a recombinant-antigen sandwich ELISA for improved antibody detection against *Rickettsia* spp. Three *Rickettsia akari* proteins, rGroEL, rDnaK, and rA8GP63 (uncharacterized protein), were produced and validated for immunogenicity. The assay was evaluated using 94 patient serum samples, including those with positive, negative, and unknown clinical course. The optimized ELISA demonstrated high reproducibility, with IgG sensitivity of 89.47–95.39% and specificity of 90%. IgM detection, also assessed, showed lower sensitivity (42.11–82.89%) but maintained strong specificity (83.33%). The diagnostic performance was comparable to that of a commercial indirect immunofluorescence assay, with no cross-reactivity detected in sera from patients with unrelated infections. rDnaK and rA8GP63 represent newly explored diagnostic candidates. These findings highlight the potential of this recombinant protein-based ELISA as an accessible, sensitive and specific diagnostic tool, with a meaningful clinical impact for improving the early and accurate detection of rickettsial infections.

## 1. Introduction

Rickettsiosis is a group of infectious diseases caused by *Rickettsiae*, which are obligate intracellular bacteria belonging to the class Alpha-proteobacteria and the order *Rickettsiales*. These pathogens are maintained in nature through complex transmission cycles involving mammalian reservoirs and hematophagous arthropod vectors, such as ticks, mites, fleas, and lice, which facilitate bacterial transmission to humans via their bites or by contaminating small skin injuries with infectious arthropod feces [1,2,3].

Rickettsial diseases share common clinical signs, including fever, headache, and malaise, and sometimes are accompanied by a maculopapular, vesicular, or petechial rash localized at the site of an arthropod bite [4]. However, the disease occasionally presents non-specific clinical manifestations, which can complicate timely and accurate diagnosis. These limitations contribute to frequent delays in diagnosis and under-recognition of rickettsial infections [5,6,7].

Diagnostic options for rickettsiosis are currently limited. Although molecular assays permit early detection of infection, their high costs, technical complexity, and variable sensitivity restrict their widespread use, particularly in endemic regions [6,8,9,10,11]. Serological techniques, while more accessible, typically detect antibodies only after 7 to 10 days post-infection and are often compromised by cross-reactivity and lack of species specificity. Among these methods, immunofluorescence assay (IFA) is considered the gold standard for diagnosis due to its high sensitivity and specificity in detecting rickettsial antibodies, allowing confirmation of infection [8,9]. However, a definitive diagnosis often requires a fourfold rise in antibody titers between acute and convalescent sera [7]. In addition to IFA, enzyme-linked immunosorbent assay (ELISA) serves as a practical diagnostic test that is more accessible and easier to standardize than IFA. However, its sensitivity can vary significantly depending on the antigen selected, as different antigens may not be equally recognized by all patient antibody responses [6]. The development and use of recombinant proteins as antigens in serological assays have improved specificity and reduced cross-reactivity, enhancing diagnostic accuracy for specific *Rickettsia* species [7]. Furthermore, the Sandwich-ELISA is a well-established technique characterized by high sensitivity and specificity for detecting bacterial proteins in infectious diseases [12], including those caused by *Rickettsia* species. This assay employs a dual-antibody format consisting of a capture and a detection antibody, which minimizes nonspecific interactions and enables quantitative analysis via colorimetric signal detection [13].

In this work, we focused on the development and standardization of a novel in-house sandwich ELISA using recombinant proteins from R. akari to detect rickettsial antibodies in human serum. For this purpose, we overexpressed and purified two selected immunogenic proteins—rDnaK and rGroEL, which are conserved in all *Rickettsia* spp.—and one uncharacterized protein (Uniprot: A8GP63). The selection of the three recombinant proteins was based on findings from a previous proteomic analysis of *R. akari* [14], which demonstrated their high immunoreactivity with patient sera. The 60 kDa chaperonin GroEL and 70 kDa chaperonin DnaK were chosen because they are highly conserved heat shock proteins and known immunodominant antigens across multiple Rickettsia species, making them suitable targets for general serodiagnosis. The A8GP63 was selected as a potential marker as it was previously described as an outer membrane protein candidate. Our results were comparable to those obtained with a commercial indirect IFA kit (Vircell, Spain), suggesting that our sandwich ELISA is a potential serological assay for the reliable and accurate detection of rickettsial antibodies.

## 2. Materials and Methods

### 2.1. Preparation of Recombinant Antigens

Based on the findings of a previous study [14], we successfully expressed three recombinant (rGroEL [Uniprot: A8GPB6], rDnaK [Uniprot: A8GMF9], and uncharacterized protein [Uniprot: A8GP63]) antigens, derived from *R. akari* (strain Hartford) in the *E. coli BL21(DE3)* bacterial system. These antigens were purified using the His-TALON affinity purification kit (Takara, Beijing, China) according to the manufacturer’s instructions, and concentration was measured by the Bicinchoninic acid kit (BCA) (Pierce, Thermo Fisher, Rockford, IL, USA). Their purity and immunoreactivity were analyzed by sodium dodecyl sulfate-polyacrylamide gel electrophoresis (SDS-PAGE) and Western blotting. Samples were analyzed using MALDI TOF mass spectrometry (50–1950 *m*/*z* range) (Bruker, Billerica, MA, USA). Protein identification was performed using MASCOT v3.0 (PLGS, Waters, Milford, MA, USA) against the *Rickettsiaceae* proteome database. Matches were considered valid if they had a high score and a sequence identity of ≥15%.

### 2.2. Mouse and Human Sera Samples

Polyclonal mouse sera were obtained according to our previous work [14]. Furthermore, these anti-rGroEL, anti-rDnaK, and anti-rA8GP63 polyclonal mouse sera were subjected to IgG enrichment by polyethylene glycol (PEG) according to an earlier study [15]. This enrichment step was performed to enhance the purity and specificity of the capture antibodies for the subsequent ELISA. Briefly, 0.47 g of PEG 8000 (Sigma-Aldrich, St. Louis, MO, USA) was diluted in 1 mL of phosphate-buffered saline (PBS) at pH 7.2. The resulting solution was then added to 1 mL of mouse serum in a dropwise manner and gently vortexed until PEG was completely dissolved. Additionally, the solution was incubated at 4 °C for four hours (h), and centrifuged for 30 min at 2400× *g*. The supernatant was decanted, and the precipitate was dissolved in PBS (pH 7.2). Purity and concentration of each enriched polyclonal sera were determined by SDS-PAGE and BCA Protein Assay Kit, respectively.

All experimental animal work was performed in the experimental animal facility at the Biomedical Research Center of the Slovak Academy of Sciences (BMC, SAS) under protocol RO-2977-3/2020-220 and approved by the ethical committee of BMC, SAS.

A total of 94 human serum samples were obtained from the serum collection of the Department of Rickettsiology, Biomedical Research Center of SAS in Bratislava, which also operates as the Executive Laboratory of the National Reference Center for Rickettsioses (Regional Authority of Public Health in Banská Bystrica, Slovakia).

### 2.3. Western Blotting

Initially, 2 μg of each purified protein was separated on a 12% SDS-PAGE gel and transferred to PVDF membranes (Pall Life Sciences, Port Washintong, NY, USA) using 100 V for 90 min at 4 °C. Membranes were blocked overnight at 4 °C with 5% non-fat dry milk, followed by three 10 min washes in Tris-buffered saline with 0.05% Tween 20 (TBS-T). Then, pre-cleaned polyclonal mouse antibodies generated for each protein, diluted 1:1000 in 2.5% non-fat milk in TBS, were applied to the membrane for 1 h at room temperature (RT). After three washes, HRP-conjugated goat anti-mouse IgG (Thermo Fisher Scientific, Rockford, IL, USA), diluted 1:3000 in 2.5% non-fat milk in TBS, was added and incubated 1 h at RT. Later, the membranes were washed five times with TBS-T, and detection was performed using the electrochemiluminescence (ECL) technique and X-ray film exposure (Thermo Fisher, USA).

### 2.4. Indirect Immunofluorescence Assay (IFA)

The commercial IFA kit from Vircell (Granada, Spain) was used for the validation of our in-house sandwich ELISA. This test is capable of detecting Rickettsia anti-IgM and anti-IgG antibodies in human serum. All the samples were analyzed according to the manufacturer’s instructions. The analysis of slides was performed on a Nikon Eclipse DS Qi2 fluorescence microscope (Nikon, Tokyo, Japan) at 400× magnification. Positive and negative controls from patients were previously tested in the laboratory routine. The dilution cut-off value for patient sera was at 1:128 for IgM and 1:256 for IgG.

### 2.5. Optimization of Sandwich ELISA Method with Recombinant Proteins for Identification of Rickettsial Antibodies

Our recombinant sandwich ELISA was optimized as follows. A checkerboard was made with different concentrations of the rickettsial recombinant proteins (at concentrations of 0, 1, 1.5, 2, 2.5, 3, 4, 5 µg/well) in combination with enriched polyclonal mouse sera generated for each protein at a concentration range from 50 µg/well to 0.1 µg/well. The checkerboard was performed on different plates containing various surfaces, including hydrophilic (MaxiSorp, Thermo Scientific, Roskilde, Denmark), slightly hydrophilic (MediSorp, Thermo Scientific, Roskilde, Denmark), very hydrophilic (MultiSorp, Thermo Scientific, Roskilde, Denmark), and hydrophobic (PolySorp, Thermo Scientific, Roskilde, Denmark) types. Among these, the hydrophobic PolySorp plates yielded the best balance between coefficient of variation (CV) and signal-to-noise (S/N) and were thus selected for further assay. It was found that 1 µg/well of each polyclonal mouse serum was sufficient to yield a strong signal with 1.5 µg/well of the corresponding rickettsial recombinant proteins. These combinations achieved an S/N ratio of 3.64, whereas alternative concentrations failed to reach this threshold, with a maximum S/N ratio of only 2.67. The dilution of the HRP-conjugated rabbit anti-human IgG or IgM antibodies (Dako-Aligent, Carpinteria, CA, USA) used as detector antibodies was optimized following the same strategy. A robust signal was observed at dilution 1:3000 for IgG detection and 1:1000 for IgM. Assay efficiency was assessed using S/N ratios, with acceptance criteria that included a duplicate CV of less than 10%. The CV was calculated as the standard deviation divided by the mean of two replicate measurements, and expressed as a percentage. Precision was evaluated using 12 positive controls (PCs), 5 negative controls (NCs), and 10 patient-derived samples. Each sample was analyzed in two replicates and repeated across five independent runs to quantify intra-assay and inter-assay variability.

After optimization, the sandwich ELISA was applied to detect rickettsial antibodies in human patient sera using specific rickettsial recombinant proteins. For this assay, 1 µg/well of enriched polyclonal mouse antibody (anti-rGroEL, anti-rDnaK, or anti-rA8GP63) serving as the capture antibody, was diluted in 50 µL/well of carbonate buffer (50 mM Na_2_CO_3_, 50 mM NaHCO_3_, pH 9.6) and coated separately onto individual wells of 96-well PolySorp microplates (Thermo Scientific, Roskilde, Denmark), followed by incubation at 4 °C overnight. As blanks, the selected wells were filled with buffer only. The wells were washed 3 times with 300 µL of PBS-T (8 g NaCl, 2.9 g Na_2_HPO_4_, 0.2 g KCl, 0.2 g KH_2_PO_4_, 500 μL Tween 20, and up to one liter of distilled water) and supplemented with 100 µL of blocking buffer (bovine serum albumin-BSA [Sigma-Aldrich, St. Louis, MO, USA] at 5% in PBS-T) at 37 °C for 1 h. After washing the wells with PBS-T, recombinant protein (1.5 µg/well) was added separately to individual wells and incubated at 4 °C for 18 h. Subsequently, the microplate was washed with PBS-T to remove any traces of unbound recombinant protein. To prevent non-specific binding, 100 µL of blocking buffer was applied to the wells and incubated at 37 °C for 1 h, followed by three washes with PBS-T. Fifty microliters of patient sera diluted 1:100 in 2.5% casein solution containing 1.7% BSA was added to the plate and incubated at 37 °C for 1 h. The plate was washed as above, and 50 µL/well of HRP-conjugated polyclonal rabbit anti-human IgG (1:3000) or IgM (1:1000) was added, followed by incubation at 37 °C for 1 h. After the final wash, the detection step was performed by adding 50 µL/well of freshly prepared 0.05% 1,2-phenylenediamine dichloride (Sigma-Aldrich, USA) dissolved in citrate buffer (pH 5.0) containing 0.01% hydrogen peroxide as the substrate, followed by incubation at 37 °C for 20 min. The reaction was stopped by the addition of 50 µL 2.3 M H_2_SO_4_. Absorbance was read at 490 nm (OD490) using an ELx808 microplate reader (BioTek, Winooski, VT, USA). The cut-off value was determined by receiver operating characteristic (ROC) curve analysis, which identified the point on the curve that offers the best balance between sensitivity and specificity. The OD value corresponding to this point served as the cut-off; sera with an OD below it were classified as negative. All of the recombinant proteins and capture antibodies described above were used under identical conditions and at equivalent concentrations. Cross-reactivity was evaluated with 23 patient sera positive for *Coxiella burnetii*, *Chlamydia* sp., SARS-CoV-2, and tick-borne encephalitis.

### 2.6. Avidity Assay

To evaluate the binding strength of recombinant proteins to human antibodies, an avidity assay was performed following established protocols [16,17], with modifications of ammonium thiocyanate (AT) concentration and incubation time. Briefly, a sandwich ELISA was conducted, incorporating an additional dissociation step. Following the incubation of patient serum and subsequent washes, 50 µL of AT (0.1–5 M in PBS) was added to each well and incubated for 15 min at either RT or 37 °C. Plates were then washed four times with PBS to remove unbound complexes, after which the assay proceeded as previously described. Optimal assay conditions were determined by analyzing 5-parameter logistic (5PL) regression curves generated for each positive control. The inflection point of the curve was used to identify the optimal condition, which was established as 0.5 M AT at RT for 15 min.

### 2.7. Data Processing and Statistical Analysis

ELISA data were analyzed using GraphPad Prism 5.03 and Microsoft Excel. For the sandwich ELISA, duplicate optical density (OD) values were averaged, and standard curves were fitted using a 5PL model. Assay repeatability was assessed by calculating the standard deviation (SD) and CV from control samples and ten patient sera. Diagnostic performance was determined through ROC analysis, with sensitivity, specificity, and diagnostic odds ratio (DOR) derived from cut-off points established by the Youden index. The DOR values for each assay were calculated using the formula:

$$DOR=\frac{sensitivity\times specificity}{\left(1-sensitivity)\times (1-specificity\right)}$$

Agreement between IFA operators was assessed with Cohen’s Kappa, whereas comparability between IFA and sandwich ELISA was evaluated using Chi-square tests. The avidity index (AI) was calculated following the formula:

$$AI=\frac{OD\;of\;washed\;AT-treated\;well}{OD\;of\;washed\;buffer-treated\;well}$$

as previously described [16,17]. Mean AI values for PC, NC, and patient sera were compared using a one-way analysis of variance (ANOVA).

## 3. Results and Discussion

Here, we addressed the need for an improved serological method to detect rickettsial antibodies in patient samples by systematically optimizing assay parameters sequentially, resulting in the development of a sandwich ELISA for IgM and IgG detection in human serum samples. The performance of this assay was subsequently evaluated by comparison with the commercial indirect IFA, which is widely regarded as the gold standard for the diagnosis of rickettsiosis.

**Figure 1:**
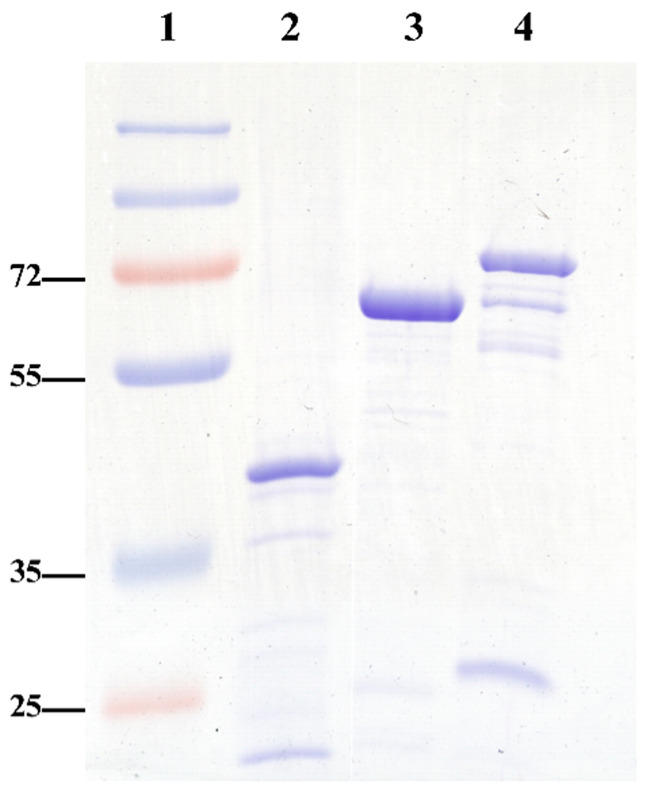
Visualization of purified recombinant proteins. 10 µg of each purified recombinant protein was separated in 12% SDS-PAGE and stained with Coomassie Brilliant Blue. Lane 1: Page Ruler Plus Prestained Protein Ladder (Thermo Scientific, USA); Lane 2: rA8GP63; Lane 3: rGroEL; Lane 4: rDnaK.

An initial optimization step involved the production of recombinant antigens, specifically recombinant proteins (rGroEL, rDnaK, and rA8GP63), prepared as previously described [14]. Following purification, the proteins were concentrated using Amicon ultrafiltration devices. Protein purity and integrity were assessed by SDS-PAGE, followed by Coomassie Brilliant Blue staining. Prominent bands on the gel corresponded to the expected molecular weights, along with minor bands that likely represent protein oligomers or degradation products (Figure 1). Additionally, densitometric analysis of the SDS–PAGE bands was performed using ImageJ version 1.53q (analysis software). Each recombinant protein appeared as a strong, well-defined band with minimal background, indicating good enrichment. Integrated optical density values (7.175 for rA8GP63, 7.549 for rGroEL, and 9.612 for rDnaK) showed that the target proteins contributed most of the signal in their respective lanes, confirming high relative purity. The similar intensities of rA8GP63 and rGroEL suggest consistent expression and recovery across preparations, whereas rDnaK exhibited the highest yield. Densitometry was used only for relative comparison, and absolute protein concentrations were determined independently by BCA assay. The average yields obtained were 11.4 mg of rGroEL, 17.2 mg of rDnaK, and 9.6 mg of rA8GP63 per liter of *E. coli BL21(DE3)* culture.

**Figure 2:**
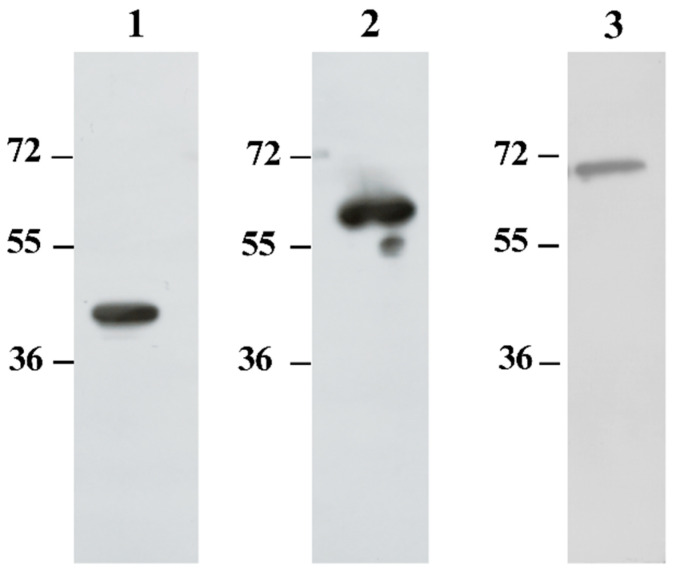
Immunogenicity of recombinant Rickettsial proteins. Purified recombinant proteins were analyzed by Western blot using polyclonal mouse sera raised against each recombinant protein. Lane 1: rA8GP63; lane 2: rGroEL; lane 3: rDnaK.

Immunogenicity of the recombinant proteins was evaluated by Western blot using sera from BALB/c mice immunized with each protein. All three recombinant proteins elicited strong IgG reactivity in murine sera at a 1:1000 dilution (Figure 2). Due to the robust reactivity of mouse sera against the recombinant proteins, these sera were subsequently utilized as capture antibodies in the development of our sandwich ELISA.

Following optimization of assay conditions, 94 patient serum samples were analyzed to assess the diagnostic performance of the sandwich ELISA. All recombinant antigens exhibited low intra-assay variability, confirming the robustness of the optimized protocol. For IgG detection, the mean CVs were 6.24% for rA8GP63, 7.03% for rGroEL, and 7.96% for rDnaK. Similarly, low variability was observed for IgM detection, with mean CVs of 6.23% for rA8GP63, 6.52% for rGroEL. Notably, rDnaK exhibited the lowest CV for IgM detection (4.36%), suggesting particularly stable performance for this antigen–antibody interaction.

**Figure 3:**
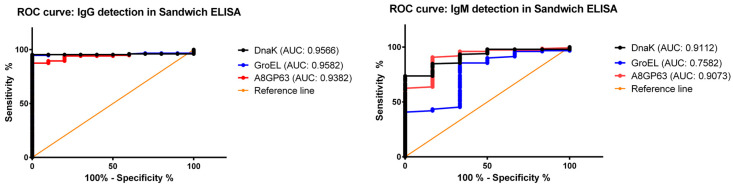
Receiver operating characteristic curve (ROC) of recombinant rickettsial proteins in sandwich ELISA. Area under the curve (AUC) values exceeded the baseline of 0.7 for both IgG and IgM detection.

Given incomplete clinical histories for some specimens, ROC analysis provided an objective assessment of assay sensitivity and specificity [18,19,20]. The Area Under the Curve (AUC) obtained from ROC analyses was used to assess the alignment between the sandwich ELISA results and clinical diagnoses, using sera from patients with confirmed rickettsiosis as reference standards. As illustrated in Figure 3, all assay formats achieved AUC values above 0.7, reflecting strong diagnostic agreement and demonstrating the overall robustness of the developed sandwich ELISA approach. Notably, IgG detection showed particularly high diagnostic accuracy, with AUC values of 0.9566 for rDnaK, 0.9582 for rGroEL, and 0.9382 for rA8GP63. IgM detection using rDnaK (0.9112) and rA8GP63 (0.9073) also yielded high AUC values, supporting their reliability for clinical application. In contrast, IgM detection with rGroEL produced a lower AUC of 0.7582, indicating reduced diagnostic performance for this protein in IgM-based assays. In accordance with the AUC values, the assay also exhibited strong sensitivity and specificity for detecting IgG and IgM antibodies. The assay sensitivity was reduced for IgM detection using the rGroEL (42.11%) and rA8GP63 (67.11%) antigens, which is consistent with previous studies reporting limited reliability of IgM-based assays in rickettsial diagnostics [8]. However, specificity across all tested proteins remained high (80%), indicating low false positivity rates (Table 1).

Moreover, we utilized confidence intervals (CIs) to assess the reliability of our diagnostic performance estimates. A CI defines the range of values that is likely to contain an unknown population parameter, such as the true sensitivity or specificity of our assay [21,22]. As shown in Table 1, all calculated parameters, sensitivity, specificity, and DOR, fall within the established CIs, which were calculated using a confidence level of 95%. This means that if the study were repeated many times, 95% of the resulting confidence intervals would contain the true population values for these diagnostic metrics. This finding further supports the statistical power and robustness of the reported assay parameters.

The DOR value serves as a robust and unbiased measure of diagnostic effectiveness by combining sensitivity and specificity into a single metric and is not influenced by disease prevalence [23]. Analysis revealed that all recombinant proteins exhibited high specificity; however, DOR values were significantly lower for IgM compared to IgG detection, indicating reduced overall diagnostic accuracy in IgM-based assays. This discrepancy is attributed to the inherent properties of IgM antibodies, which have lower binding affinity and specificity, leading to an increased risk of false negatives and diminished assay reliability [8]. These findings highlight the importance of prioritizing IgG detection in serological diagnostics for rickettsial infections to ensure optimal sensitivity and specificity.

The limited clinical metadata available for the specimens necessitated the reliance on objective ROC curve analysis for assay assessment [24,25]. While ROC analysis provides a robust mathematical measure of assay performance, the lack of a complete clinical history (e.g., precise time of symptom onset or confirmation of primary versus recurrent infection) prevents a detailed, phased evaluation of antibody kinetics. Therefore, to ensure the assay’s clinical utility and generalizability, larger, geographically diverse validation studies are necessary. Such expanded validation, incorporating samples from different endemic regions and specimens with well-defined time points relative to symptom onset, is crucial for fully clarifying the IgM kinetics, determining the assay’s utility across varying rickettsia species, and establishing reliable diagnostic cut-offs for a broader population.

Assay validation included a comparative analysis between our developed sandwich ELISA and the reference indirect IFA using the Chi-square test (Table 2). No significant differences were observed in overall positivity rates between the sandwich ELISA format and IFA (*p*-values > 0.05), indicating strong agreement between the two diagnostic modalities. Specificity assessment and cross-reactivity exclusion involved testing 23 sera from patients with unrelated infectious diseases (Q fever, Tick-borne encephalitis, COVID-19, and *Chlamydia*). The OD values for these samples ranged from 0.06 to 0.24, all below the established diagnostic cut-off, confirming the absence of cross-reactivity. This specificity stems from the fact that, although GroEL and DnaK are evolutionarily conserved heat shock proteins, they retain species-specific epitopes that differ significantly from those found in control pathogens, which belong to different bacterial and viral classes. These findings demonstrate the assay’s ability to reliably distinguish rickettsial infection from other febrile illnesses, minimizing false-positive results.

Antibody avidity testing was performed using all three recombinant proteins to evaluate the stability and specificity of antigen–antibody interactions (Table 3). The AI for IgG and IgM were determined using a chaotropic agent-based ELISA, wherein serum samples were subjected to AT treatment to disrupt low-affinity interactions. Statistical analysis revealed no significant differences in AI for either IgG or IgM between PC, NC, and patient samples (*p* > 0.05). These results indicate that the recombinant antigen–antibody complexes formed during the assay exhibit consistent stability and specificity, thereby supporting the robustness and diagnostic accuracy of the sandwich ELISA developed for detecting rickettsial infections.

To our knowledge, DnaK has not previously been applied in diagnostic ELISA formats for humans. The recombinant GroEL, however, has been extensively studied for this purpose. Earlier investigations employed GroEL-based ELISAs to detect *Bartonella henselae*-specific IgM and IgG, reporting specificities of 97.7% for IgM and 82.0% for IgG, and sensitivities of 45.3% and 42.9%, respectively [24]. Our results are consistent with these findings, but they also show improved IgG sensitivity. Similar studies have demonstrated that GroEL effectively distinguishes chronic from acute *Coxiella burnetii* infections, with all chronic-phase samples exhibiting OD values above the diagnostic cut-off [25]. Within the *Rickettsia* genus, GroEL has been validated in previous research on *R. typhi* and *R. conorii*, where it produced moderate likelihood ratio values yet remained diagnostically valuable [26]. Our analysis further highlights rGroEL as one of the top-performing antigens, reinforcing its broad utility in serological diagnostics of intracellular pathogens.

## 4. Conclusions

In this study, we addressed the critical need for an improved method to detect rickettsial antibodies in human sera. Three recombinant proteins (rA8GP63, rGroEL, and rDnaK) were expressed in *E. coli* and purified, preserving their native epitopes. The proteins elicited strong IgG and IgM responses, confirming their diagnostic potential. Utilizing these recombinant antigens, a novel sandwich ELISA was developed that demonstrated high sensitivity, specificity, and diagnostic accuracy for both IgG and IgM detection, with strong agreement compared to the commercial indirect IFA. No cross-reactivity was observed with unrelated infections. rDnaK and rA8GP63 emerged as novel markers, while rGroEL further validated its diagnostic role. Despite several limitations. this assay offers a scalable and reliable alternative to current Rickettsia diagnostic methods.

## Figures and Tables

**Table 1:** Statistical evaluation of the diagnostic performance of sandwich ELISA with recombinant proteins.

Parameter	rA8GP63 (CI)		rGroEL (CI)		rDnaK (CI)	
	IgG	IgM	IgG	IgM	IgG	IgM
Sensitivity	89.47% (83.47–93.86)	67.11% (59.03–74.5)	95.39% (90.74–98.13)	42.11% (34.15–50.37)	95.39% (90.74–98.13)	82.89% (75.95–88.51)
Specificity	90.00% (55.5–99.75)	83.33% (35.88–99.58)	90.00% (55.5–99.75)	83.33% (35.88–99.58)	90.00% (55.5–99.75)	83.33% (35.88–99.58)
DOR	74.47 (56.7–103.1)	10.2 (6.04–17.22)	186.3 (138.55–250.31)	3.64 (2.1–6.27)	186.3 (138.55–250.31)	19.87 (14.45–40.57)

CI: confidence interval.

**Table 2:** Comparative Chi-square test analysis of seropositivity results from the sandwich ELISA using recombinant proteins versus commercial indirect IFA.

Antibodies	Protein	ELISA Positive	ELISA Negative	IFA Positive	IFA Negative	Chi-Value	Significance (*p*-Value) ^†^
IgG	A8GP63	8 (8.5%)	86 (91.5%)	5 (5.3%)	89 (94.7%)	0.7437	0.3885
	GroEl	4 (4.3%)	90 (95.7%)	5 (5.3%)	89 (94.7%)	0.1167	0.7326
	DnaK	3 (3.2%)	91 (96.8%)	5 (5.3%)	89 (94.7%)	0.5222	0.4699
IgM	A8GP63	11 (11.7%)	83 (88.3%)	5 (5.3%)	89 (94.7%)	2.459	0.1168
	GroEL	10 (10.6%)	84 (89.4%)	5 (5.3%)	89 (94.7%)	1.811	0.1784
	DnaK	8 (8.5%)	86 (91.5%)	5 (5.3%)	89 (94.7%)	0.7437	0.3885

^†^ *p*-value < 0.05 was considered statistically significant.

**Table 3:** Assessment of the stability and specificity of antigen–antibody binding interactions.

Antibodies	Protein	Positive Control AI Mean (±SD)	NC AI Mean (±SD)	AI Mean (±SD) Samples	F-Value (Statistical Difference)	*p*-Value (Significance) ^†^
IgG	A8GP63	0.398 ± 0.03	0.26 ± 0.02	0.495 ± 0.052	0.09236	0.913
	GroEL	0.415 ± 0.027	0.26 ± 0.03	0.515 ± 0.073		
	DnaK	0.4 ± 0.028	0.24 ± 0.02	0.43 ± 0.085		
IgM	A8GP63	0.73 ± 0.114	0.381 ± 0.019	0.496 ± 0.08	0.5684	0.5942
	GroEL	0.55 ± 0.1	0.364 ± 0.015	0.45 ± 0.073		
	DnaK	0.493 ± 0.015	0.327 ± 0.017	0.47 ± 0.055		

^†^ *p*-value < 0.05 was considered statistically significant.

## Data Availability

The authors confirm that the data supporting the findings of this study are available within the article.

## Abbreviations

The following abbreviations are used in this manuscript:

ELISA	Enzyme-linked immunosorbent assay
IFA	Immunofluorescence assay
BCA	Bicinchoninic acid colorimetric reaction
PEG	Polyethylene glycol
AT	Ammonium Thiocyanate
CV	Coefficient of variation
P/N	Positive-to-Negative ratio
OD	Optical Density
5PL	Five-parameter logistic model
SD	Standard Deviation
S/N	signal-to-noise ratio
ROC	Receiver Operating Characteristic
DOR	Diagnostic Odds Ratio
AI	Avidity Index
